# Yeast as a Tool to Study Signaling Pathways in Mitochondrial Stress Response and Cytoprotection

**DOI:** 10.1100/2012/912147

**Published:** 2012-02-02

**Authors:** Maša Ždralević, Nicoletta Guaragnella, Lucia Antonacci, Ersilia Marra, Sergio Giannattasio

**Affiliations:** CNR—Istituto di Biomembrane e Bioenergetica, Via Amendola 165/A, 70126 Bari, Italy

## Abstract

Cell homeostasis results from the balance between cell capability to adapt or succumb to environmental stress. Mitochondria, in addition to supplying cellular energy, are involved in a range of processes deciding about cellular life or death. The crucial role of mitochondria in cell death is well recognized. Mitochondrial dysfunction has been associated with the death process and the onset of numerous diseases. Yet, mitochondrial involvement in cellular adaptation to stress is still largely unexplored. Strong interest exists in pharmacological manipulation of mitochondrial metabolism and signaling. The yeast *Saccharomyces cerevisiae* has proven a valuable model organism in which several intracellular processes have been characterized in great detail, including the retrograde response to mitochondrial dysfunction and, more recently, programmed cell death. In this paper we review experimental evidences of mitochondrial involvement in cytoprotection and propose yeast as a model system to investigate the role of mitochondria in the cross-talk between prosurvival and prodeath pathways.

## 1. Introduction

When faced with stressful conditions, cells display a molecular response which either allows them to adapt and survive or, alternatively, cause cell demise. Depending on the level and mode of stress, different mechanisms have been described for a number of modes in which cells die. The concept of cellular demise and associated terminology have been evolving since the first characterization of apoptosis [1] as a form of programmed cell death (PCD), as opposed to an accidental mode of cell death, that is, an unregulated process termed necrosis. Mounting evidence has been collected in the last three decades of the existence of at least three major types of regulated cell death mechanisms, depending on cell type and death stimulus: type I, or apoptosis, that is of critical importance for the survival of multicellular organisms, being involved in tissue homeostasis, embryonic development, and in the immune response, type II, or autophagy, an important multifunctional process whose main function is recycle of cellular constituents, and type III, or necrosis (necroptosis), which occurs after the exposure to severe stressful conditions ([2, 3] and references therein). These mechanisms do not exclude each other and conditions which block, for example, apoptosis, may cause autophagy or necrosis induction [4].

However, under certain conditions, cells can respond to stress by activating prosurvival strategies leading to cytoprotection. The most common pro-survival strategies reported involve direct antagonizing of proapoptotic proteins and increase in the expression levels of antiapoptotic genes (see [5]). Another cytoprotective strategy adopted by cells is preconditioning, which has been extensively studied in the heart and the brain cells. It has been shown that brief sublethal periods of ischemia/reperfusion induce anti-apoptotic phenotype in these cells, protecting them from ischemia/reperfusion mediated death [6–8]. Actually, preconditioning with any apoptotic-inducing agent confers the cells with cytoprotective phenotype. It appears that this cytoprotective strategy is mediated by the increased expression of a group of powerful anti-apoptotic genes, such as genes encoding reactive oxygen species (ROS) scavengers, heat shock proteins (HSPs), and other chaperones [9]. 

Several molecular pathways causing cell adaptation have been characterized, depending on the type of stress impinging on the cell: (i) heat-shock response, (ii) unfolded protein response, (iii) DNA damage response, (iv) response to oxidative stress, (v) autophagy. Although both anti-apoptosis and cell survival pathways serve to counteract PCD, these pathways are mechanistically distinct from the processes that regulate cell death and there is cross-talk among them [9].

For example, one of the major modes in which cells counteract a stress to remain alive is the activation of autophagy promoting cell survival. Autophagy is a genetically regulated bulk degradation program conserved from yeast to humans, in which long-lived proteins and damaged organelles are delivered to lysosomes in mammals or to the vacuoles in yeast, where they are degraded and their components recycled. The canonical autophagic pathway has been elucidated at the molecular level, and the autophagy-related (ATG) genes were first identified in yeast, with homologous found in all eukaryotes [10]. The contribution of such pathway to autophagic cell death (type II PCD) as well as the molecular mechanisms by which autophagy is activated as pro-survival or prodeath cell stress response are yet unknown. There is evidence of cross-talk between apoptosis and autophagy at the molecular level through antiapoptotic B-cell lymphoma-2 (Bcl2) family proteins [11].

A detailed description of all the modes of cell adaptation and demise in response to stress conditions is beyond the scope of this paper. Readers are referred to comprehensive reviews on the topics [5, 9]. Here, we will rather focus on the role of mitochondria in cell stress response and cytoprotection.

## 2. Mitochondrial Pathways in Cell Deathand Survival

Besides its well-recognized bioenergetic function, mitochondria play an important role in many cell regulatory and signaling events, including apoptosis [12]. Two main apoptosis signaling pathways have been delineated: the extrinsic and intrinsic or mitochondrial pathway [13]. The extrinsic pathway is mediated by a subgroup of tumor necrosis factor receptors (TNFR) superfamily, so-called death receptors. Binding of a ligand induces receptor clustering and formation of a death-inducing signaling complex (DISC) (see [13]). Via the Fas-associated death domain protein (FADD), the adaptor molecule, this complex recruits multiple procaspase 8 molecules, resulting in the activation of caspase 8, one of the members of a family of cysteine proteases that function as common death effector molecules [14]. The intrinsic pathway is regulated mainly by mitochondria. Internal insults cause mitochondrial dysfunction, that is, energetic failure, oxidative stress, lipid metabolism abnormalities, and apoptosis sensitization. It involves stress-mediated release of cytochrome c (cyt c), which then binds to apoptotic protease activating factor 1 (Apaf-1), procaspase 9 and possibly other molecules to form apoptosome, which causes activation of caspase 3 (an effector caspase). Pro- and anti-apoptotic Bcl-2 proteins compete to regulate cyt c release from mitochondria. Apart from the cyt c, other apoptogenic factors are released from mitochondria as well, such as apoptosis-inducing factor (AIF), endonuclease G, Omi/high-temperature requirement protein A (HtrA2), or second mitochondria-derived activator of caspase (Smac)/direct inhibitor of apoptosis proteins (IAP) binding protein with low PI (DIABLO). Mitochondrial membrane permeabilization is a decisive event in the execution of apoptosis and the associated bioenergetic deficiency is usually irreversible and commits cells to die [15]. Activation of either apoptotic pathway leads eventually to proteolytic degradation of cellular components by caspases. Cross-talk between the two pathways is provided by Bid, a pro-apoptotic member of Bcl-2 protein family, activated by caspase 8 (see [16]).

Alternatively, in certain conditions, cells can respond to mitochondrial stress by adaptation, thus ensuring cells survival. Processes that lead to cytoprotection through resistance to apoptosis are probably best characterized in tumor cells, given the altered regulation of the apoptotic process during tumorigenesis. Upregulation of anti-apoptotic genes, such as Bcl-2 and FLICE-like inhibitory protein (c-FLIP), as well as a decrease in the expression of pro-apoptotic genes, such as Bax, involved in mitochondrial permeabilization, are likely the most common mechanisms involved in development of anti-apoptotic phenotypes in cancer cells [17].

Dormancy stages, such as hibernation and diapauses, or spore stages in yeast, are examples of survival strategies under long-term stressful environmental conditions. Some animals, like embryos of the brine shrimp, *Artemia franciscana*, can survive under anoxic conditions at room temperature for years [18], avoiding apoptotic or necrotic cell death, possibly by the absence of a regulated mitochondrial permeabilization and cyt c release [19].

Several lines of evidence suggest that the primary mechanism to eliminate dysfunctional, aged or excess mitochondria, promoting cell survival, is the selective form of autophagy, termed mitophagy [20, 21]. It has also been shown that the mitochondrial stress responses (MSRs) are mediated through the activity of the transcriptional coactivator peroxisome-proliferator-activated receptor coactivator-1 (PGC-1)*α* [22]. PGC-1*α* activates a large number of genes involved in respiration, oxidative metabolism, and uptake and utilization of energy substrates [23].

Finally, it is of note that many cell death proteins, including caspases, AIF, endonuclease G, and serine protease Omi/HtrA2 and cyt c, besides their pro-apoptotic functions, have important roles in cellular homeostasis [24]. Activation of caspases is shown to be necessary for processes such as terminal differentiation, activation, proliferation, and cytoprotection [25]. Caspase-independent death effectors, such as AIF and Omi/HtrA2, are released from the mitochondrial intermembrane space upon apoptosis induction, but these proteins also have important functions in cellular redox metabolism and/or mitochondrial biogenesis [26–28].

Beyond these circumstantial lines of evidence of a role of mitochondria in cell decision for life and death, MSR pathways which regulate the interplay between cell adaptation and death are still poorly understood. With this respect, signaling pathways involving the role of mitochondrial calcium regulation in the cross-talk between autophagy and apoptosis have been recently reviewed [29]. 

The yeast *Saccharomyces cerevisiae* has been a preferred model organism in which major intracellular processes, such as protein synthesis, mitochondrial biogenesis, retrograde mitochondria-to-nucleus signaling pathway, the proteasome machinery, and autophagy, have been identified for the first time and characterized in more details at the molecular level. This model organism seems worth to be used in research on how cells respond to mitochondrial dysfunction, integrating death and growth signaling pathways. The best known intracellular pathways by which yeasts respond to mitochondrial dysfunctions are the programmed cell death and retrograde signaling pathways, which will be reviewed in the next paragraphs.

## 3. Programmed Cell Death in Yeast

Apoptosis-like, autophagic and necrotic cell death pathways have been detected in yeast cells exposed to different intra- and extracellular stresses. Thus, *S. cerevisiae* has been established as an ideal model system to study PCD pathways more in detail due to its easy handling and technical tractability, together with the high level of phylogenetic conservation of biochemical pathways and regulators between yeast and mammals [30].

Yeast apoptosis, which has been largely investigated in the last decade, shares most of the morphological and biochemical hallmarks of mammalian apoptosis, such as phosphatidylserine externalization to the outer layer of the cytoplasmic membrane, DNA fragmentation, chromatin condensation, ROS production, and involvement of specific pro-apoptotic proteins, including cyt c, Aif1p, and Bcl-2 homology domain 3- (BH3-) containing protein [31–33]. Mitochondrial dysfunction has also been involved in yeast PCD [34]. 

Why should a unicellular organism have developed and conserved a highly coordinated suicide program during evolution? Answers to this question are many. Microorganisms are continuously exposed to various stressors and are well known for their ability to adapt to constantly changing conditions in their surroundings through altering genome expression and metabolism [35, 36]. Apoptosis within yeast colonies can occur under physiological conditions, when it is perceived as an altruistic death of single cells that promotes the long-term survival of the whole colony [37], or it can be triggered externally by competing yeast strains or higher eukaryotes [38]. The altruistic function of yeast PCD is accomplished by eliminating infertile or otherwise damaged cells after failed mating, genetic recombinants nonadapted to the environment, and old cells during aging or development of multicellular colonies. Nonclonal enemy strains, however, can trigger death in the population by secretion of virus-encoded killer toxins, in their competition for nutrients. Higher eukaryotes, such as plants and animals, provoke apoptosis in yeast cells as their mode of defense against pathogenic fungi [39].

Apoptosis-inducing stimuli in yeast are different: chemical or physical stress, heterologous expression of human proapoptotic proteins, or endogenous triggers, such as mutations in genes involved in signal transduction pathways.

Acetic acid, produced normally by fermentation in *S. cerevisiae* cells, is one of the compounds commonly used to induce apoptosis in yeast [40]. Molecular mechanisms and cell components of yeast PCD induced by acetic acid (AA-PCD) together with acetic acid-stress adaptive response have been characterized in detail (see [41]). In this paper we propose that AA-PCD is a suitable model system to study the role of mitochondrial pathways in cell stress response. To this aim, here we make an overview on recent achievements in the mechanisms of yeast AA-PCD and focus on investigations on cytoprotective mechanisms at the mitochondrial level.

**Figure 1:**
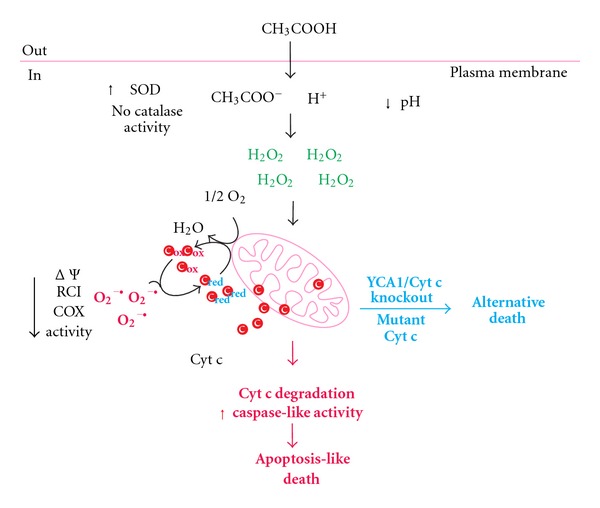
AA-PCD pathways. Acetic acid enters in yeast cells and dissociates into acetate and protons causing intracellular acidification. H_2_O_2_ accumulates, SOD activity increases, while catalase activity is undetectable. *En route* to AA-PCD, cyt c is released and works as an electron donor (c_red_) to mitochondrial respiratory chain and as superoxide anion (O_2_^−•^) scavenger (c_ox_). In a late phase, cyt c is degraded by unidentified proteases. Mitochondrial functions progressively decline with decrease in mitochondrial membrane potential (ΔΨ), respiratory control index (RCI) and COX activity. Caspase-like activity increases in a late phase (red lines). Alternative programmed cell death is induced by acetic acid in YCA1 and/or cyt c knockout cells or in mutant cyt c cells (blue lines).

The molecular mechanisms in yeast AA-PCD have been characterized in some details and are depicted in Figure 1. Time course of events after the induction of AA-PCD has been defined in a work with exponentially growing *S. cerevisiae* W303-1B cells, in which the minimum acetic acid concentration sufficient to cause cell death was 80 mM [40, 42]. Progressive loss of cell viability is complete after 200 min after AA-PCD induction. Correspondingly, AA-PCD cells showed chromatin condensation and intact plasma membrane; as well as DNA fragmentation, with the maximum percentage at 150 min [43–45].

The earliest event (15 min) following acetic acid challenge is ROS production, with a different role for hydrogen-peroxide and superoxide anion [46]. Hydrogen peroxide appears to be a second messenger in AA-PCD cascade of events, as also shown by AA-PCD inhibition by ROS scavenger N-acetyl cysteine [47]. Catalase and superoxide dismutase have been shown to modulate ROS level *en route* to AA-PCD [48].

Mitochondria are strongly implicated in AA-PCD, with the release of cyt c into the cytosol, the production of ROS, the reduction in oxygen consumption and in mitochondrial membrane potential as well as the loss of cytochrome c oxidase (COX) function [31, 44]. Starting at 60 min cyt c is released (with a maximum at 150 min) from intact coupled mitochondria, and can function both as an electron donor as well as ROS scavenger. Later on, AA-PCD mitochondria become gradually uncoupled, and released cyt c is degraded, possibly by yet unidentified proteases [44]. It is of interest that AA-PCD can also occur without cyt c release, but with a lower death rate compared to wild type (WT) cells, as it has been shown in cyt c knockout cells [49]. The role of cyt c in modulating AA-PCD has been further elucidated in mutant cells expressing a stable but catalytically inactive form of cyt c. The observation of an apoptotic resistant phenotype associated to a decrease in ROS production and the lack of cyt c release in this mutant suggests that mitochondrial cyt c in its reduced state modulates AA-PCD independently on its function as an electron carrier [50].

As in higher eukaryotes, proteolytic degradation of cell components is activated in yeast PCD. Yeast contains a gene encoding a metacaspase, named YCA1, which has a distant amino acid sequence similarity to mammalian caspases [51, 52]. YCA1-encoded metacaspase shows cleavage specificity different from that of caspases, since it can hydrolyse proteins after arginine or lysine residues but not after aspartate. Nonetheless, both target substrates and the precise function of the yeast metacaspase in PCD are still unknown [53]. Yeast cells lacking YCA1 gene undergo AA-PCD, with a lower rate in respect to WT cells, showing that metacaspase is dispensable for yeast apoptosis to occur. Although YCA1-dependent caspase-like proteolytic activity was shown to be induced in a late phase of AA-PCD (200 min), the role of YCA1 in this process appears to be independent of its caspase-like activity [43]. It is of note that YCA1 is implicated in cyt c release from mitochondria, but further investigations are needed to elucidate the role of YCA1 in this process [49]. 

The occurrence of AA-PCD in cells lacking YCA1 or respiratory deficient cells due to cyt c mutations, with features different from wild-type cell AA-PCD, suggests that acetic acid can activate alternative death pathways. In these alternative death pathways mitochondria seem to play different roles [54].

## 4. The Mitochondrial Retrograde Pathway in Yeast

**Figure 2:**
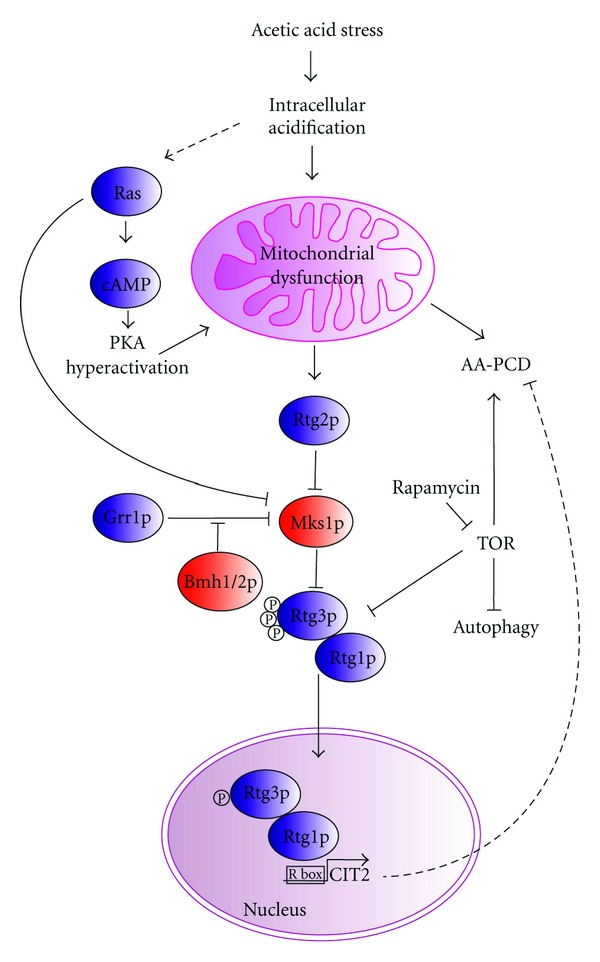
Signaling pathways possibly involved in the interplay between cell death and cytoprotection processes in yeast cells in response to acetic acid stress. Acetic acid causes intracellular acidification, mitochondrial dysfunction, and cell death. TOR pathway is involved in the signaling of AA-PCD; rapamycin inhibition of TOR can trigger autophagy and RTG target gene expression. Dotted lines indicate the hypothetical signaling pathways in AA-PCD regulation in response to mitochondrial dysfunction, on the basis of indirect experimental evidence (see text for details). Intracellular acidification stimulates RAS-cAMP signaling pathway, causing mitochondrial dysfunction, which may lead to AA-PCD or activate retrograde response inducing AA-PCD resistance. RAS pathway negatively regulates Mks1p, a negative regulator of RTG pathway. Positive and negative regulators of RTG pathway are shown in blue and red, respectively. TOR signaling is found at the crossroad of RTG, AA-PCD, and autophagy pathways.

Cells can adapt to mitochondrial dysfunctions by activating an evolutionally conserved communication pathway from mitochondria to the nucleus, termed retrograde response [55]. The best comprehension of components and molecular details of the retrograde signaling have been obtained with *S. cerevisiae* [56]. In these cells, the retrograde response leads to a reconfiguration in the expression of a subset of nuclear genes enabling accommodation to changes in the mitochondrial state. The prototypical target gene of the yeast retrograde pathway is CIT2, which encodes the peroxisomal isoform of citrate synthase functioning in the glyoxylate cycle. CIT2 expression is largely increased in cells with compromised mitochondrial functions, such as those lacking mitochondrial DNA (*ρ* ^0^) [57]. Regulation of both basal and upregulated expression of CIT2 is mainly dependent on RTG genes, encoding regulatory proteins central to yeast retrograde signaling (Figure 2). Rtg1p and Rtg3p are basic helix-loop-helix/leucine zipper (bHLH/Zip) transcription factors that interact as a heterodimer to bind target sites called R boxes (GTCAC) located in the promoter region of the retrograde target genes [58]. Activation of Rtg3p as an active transcriptional unit correlates with its partial dephosphorylation and its translocation with Rtg1p from the cytoplasm to the nucleus [59]. Crucial to this translocation is Rtg2p, a cytoplasmic protein with an N-terminal ATP-binding domain, belonging to the actin/Hsp70/sugar kinase superfamily [60, 61]. Rtg2p acts upstream of the Rtg1/Rtg3p complex, being both a proximal sensor of the mitochondrial dysfunction and a transducer of mitochondrial signals. Rtg2p regulates Rtg1/3p localization through the reversible binding with Mks1p, a negative regulator of the RTG pathway, acting in the cytoplasm downstream of Rtg2p but upstream of Rtg1/3p [62]. Mks1p promotes the phosphorylation of Rtg3p, thus inhibiting the nuclear translocation of Rtg1/3p [63, 64]. Other positive and negative regulators of the RTG pathway have been characterized, including Grr1p which mediates ubiquitination of Mks1p [61]; Bmh1p and Bmh2p, belonging to 14-3-3 protein family, which bind to Rtg3p and keep it in an inactive state [65] and Lst8p, an essential protein suggested to negatively regulate the RTG pathway with one site upstream and the other site downstream of Rtg2p [66]. The physiological role of the RTG pathway has been essentially provided by the identification of RTG-dependent target genes, including CIT2, DLD3, encoding a D-lactate dehydrogenase, and CIT1, ACO1, IDH1/2, encoding the first three enzymes in the TCA cycle [67, 68]. Thus, the RTG pathway is involved in glutamate biosynthesis, to meet the demand of nitrogen supply for biosynthetic reactions, and in mitochondrial DNA maintenance, through regulation of ACO1 [69, 70]. RTG-independent retrograde response to mitochondrial dysfunction has also been shown [71]. 

It is of note that the RTG pathway is linked to other signaling pathways, such as TOR (target of rapamycin) pathway which inhibits Rtg1/3-dependent gene expression [72]. Lst8p, a central component of the two TOR kinase complexes, TOR1 and TOR2, is suggested to have a role in connecting the RTG and TOR pathways. However, it is clear that these two pathways do not overlap but act in parallel to converge on Rtg1/3p [73]. At this regard, it has also been proposed that Aup1, a possible part of a signaling mechanism that functionally overlaps with TOR, promotes the activation of RTG pathway under mitophagic conditions [74]. The retrograde response is also related to the Ras-cAMP signaling pathway. Ras2p potentiates the retrograde response and this action is likely mediated through the negative effect of the RAS-cAMP pathway on Mks1p [75]. In terms of pro-survival and adaptive response, the RTG-dependent signaling pathway in yeast and the NF-*κ*B stress response active in mammalian cells appear to be involved in a conserved mechanism of cell stress response, further validating yeast as a model to study mitochondrial stress response pathways [76].

## 5. Cytoprotection in Yeast AA-PCD

Acetic acid-stress sensitivity of yeast cells strongly depends on the extracellular environment. Indeed, AA-PCD is induced in yeast cells growing on glucose as carbon source at pH 3.0. At neutral pH, *S. cerevisiae* cells are able to grow on acetic acid medium as the sole carbon and energy source. Under this condition, the acid is found in a dissociated form, and acetate is transported across the plasma membrane through a low-affinity electroneutral proton symport system (see [41]). The ability of *S. cerevisiae* to use acetic acid as the only carbon and energy source depends on both the utilization of acetyl-CoA in the tricarboxylic acid (TCA) cycle, mainly for ATP synthesis and biosynthetic purposes, and on the production of succinate through the glyoxylate cycle as an anaplerotic pathway [77–79]. However, acetate transport and its metabolism are inhibited under glucose repression in *S. cerevisiae *due to the activation of pathways responsible for downregulation of respiration and gluconeogenesis [80]. Moreover, at low pH, acetic acid is in the undissociated state which has been shown to penetrate cells through the plasma membrane by simple diffusion [81] facilitated by the Fps1p aquaglyceroporin channel [82]. More neutral pH inside the cell causes its dissociation into acid anions and protons, which leads to cytoplasmic acidification, thereby inhibiting important metabolic processes [83] and increasing cell toxicity. 

*S. cerevisiae* cells have been shown to be protected from AA-PCD following 30 min preconditioning in low pH medium set by HCl prior to PCD induction [42]. Adaptation is accompanied by increasing levels of both catalase and superoxide dismutase activities, together with a decrease in intracellular levels of ROS [46]. It is of note that *en route* to AA-PCD the catalase activity is undetectable [42]. Whether the catalase undergoes enzyme inactivation and/or degradation in the AA-PCD cells remains to be established. However, unlike with mouse cell lines in which autophagy occurs as a result of selective catalase degradation [84], autophagy proved to be absent in AA-PCD [85].

At low pH, acetic acid activates an adaptive response in yeast cells mediated by two mitogen-activated protein (MAP) kinases, Hog1p, involved in the high-osmolarity glycerol (HOG) signaling pathway, and Slt2p, involved in cell integrity pathway. Acetic acid adaptation is mediated by Hog1p-dependent phosphorylation of Fps1p resulting in its ubiquitination, endocytosis, and degradation, thus blocking acetic acid uptake into the cell [82]. The Hog1p-dependent degradation of Fps1p has been hypothesized as a mechanism of the protection from the AA-PCD exerted by acid preconditioning [86]. Yet, this mechanism is not likely to be active in acid-stressed preconditioned yeast cells, since acetic acid is absent in the pre-conditioning medium.

In acid-stress-adapted cells, acetic acid treatment does not cause any increase in intracellular ROS production [42]. Since ROS are mainly produced by mitochondria, a modulation in MSR may be hypothesized in this case. Figure 2 shows certain signaling pathways involved in cell response to mitochondrial dysfunction that may have a role in the cross-talk between cell death and adaptation mechanisms activated by acetic acid stress in yeast. The best characterized mechanism of response to mitochondrial dysfunction in yeast cells is the RTG pathway. RTG-dependent retrograde response to mitochondrial dysfunction seems to have a role in adaptation of *S. cerevisiae* cells to acetic acid stress, as suggested by the up-regulation of CIT2 mRNA, the prototypical RTG-target gene, in respiratory deficient cells lacking mitochondrial DNA (*ρ* ^0^), with respect to respiratory competent (*ρ* ^+^) cells, both grown in the low pH medium used for cell pre-conditioning. On the other hand, RTG pathway is inactive in conditions in which cells are sensitive to AA-PCD induction, since in this case no up-regulation of CIT2 mRNA in *ρ* ^0^ with respect to *ρ* ^+^ cells is measured (data not shown).

TOR kinase signaling pathway that regulates cell growth in response to nutrient availability has been shown to be involved in the AA-PCD [87]. Although the RTG pathway is linked to TOR signaling, which represses transcription of RTG target genes, retrograde response to mitochondrial dysfunction has shown to be separable from TOR regulation of retrograde target gene expression [73]. Note that acetic acid has been identified as an extracellular mediator of cell death during chronological aging in yeast [88, 89]. This process involves the RAS-cAMP-PKA and the SCH9 signaling pathways, which are known to control yeast cell adaptation to nutrient availability as well as chronological lifespan in yeast [90, 91]. SCH9, the yeast homologue of Akt in mammals, is a major component of TOR pathway [92]. Consistently, intracellular acidification, induced by weak acids on a low pH medium, stimulates the RAS-cAMP signaling pathway, negatively regulating cell viability [93, 94]. cAMP-PKA signaling pathway plays an important role in coordination of mitochondrial function with environmental (nutritional) changes in yeast. The inappropriate activation of PKA can lead to the production of dysfunctional, ROS generating mitochondria, and apoptosis [95].

## 6. Conclusions and Perspectives

Intracellular pathways that regulate cellular resistance to cell death are intimately connected to the regulation of cellular metabolism. Mitochondria make a crucial link between metabolic and apoptotic processes, since they have a central role in the regulation of both [96]. 

Deciphering molecules and pathways involved in fine-tuning of PCD and cytoprotective processes is of paramount importance in biomedicine, since their dysregulation lays behind the pathogenesis of diseases such as cancer. Indeed, increased cellular metabolism and apoptotic resistance are two components of cellular transformation. Mitochondrial signaling involved in the responses of cells to metabolic transitions and physiological stresses remains largely unexplored. Strong interest exists in pharmacological manipulation of mitochondrial metabolism and signaling [97].

The fermenting yeast *S. cerevisiae*, which has the ability to switch on and off respiration in response to changes in the carbon source, and tumor cells shares several features from the metabolic point of view [98, 99]. A genome-wide analysis in this model organism suggests that retrograde response to mitochondrial dysfunction plays a role in mutagenesis and genome instability and may be implicated in carcinogenesis [100]. Recent progress in the elucidation of conserved PCD pathways in the yeast *S. cerevisiae*, together with the large amount of knowledge available about cell biology and metabolism in this model organism, provides a valuable experimental tool to study the relations between PCD regulators and components of cytoprotective intracellular signaling activated by cell stress.

Limitations to the use of a unicellular model organism are present in translation into multicellular organisms or into clinical relevant conditions with modified stress responses, as in aging or diabetes. Indeed, the molecular mechanisms underlying pathological conditions faithfully recapitulated in yeast will ultimately have to be tested in human cell and animal models. Yet, understanding the complex intracellular regulatory network integrating cell adaptation and death pathways through MSR pathways in yeast is a challenge for future investigations, which will shed light on many aspects of eukaryotic cell homeostasis.

##  Authors' Contributions

M. Ždralević and N. Guaragnella contributed equally to this work.
